# Ethical and legal concerns in artificial intelligence applications for the diagnosis and treatment of lung cancer: a scoping review

**DOI:** 10.3389/fpubh.2025.1663298

**Published:** 2025-10-14

**Authors:** Ghenwa Chamouni, Filippo Lococo, Carolina Sassorossi, Nkunzi Atuhaire, Róza Ádány, Orsolya Varga

**Affiliations:** ^1^Department of Public Health and Epidemiology, Faculty of Medicine, University of Debrecen, Debrecen, Hungary; ^2^Department of Thoracic Surgery, Fondazione Policlinico Universitario A. Gemelli IRCCS, Università Cattolica del Sacro Cuore, Rome, Italy; ^3^Faculty of Medicine, Institute of Preventive Medicine and Public Health, Semmelweis University, Budapest, Hungary; ^4^HUN-REN-UD Public Health Research Group, Department of Public Health and Epidemiology, Faculty of Medicine, University of Debrecen, Debrecen, Hungary; ^5^National Laboratory for Health Security, Epidemiology and Surveillance Centre, Semmelweis University, Budapest, Hungary

**Keywords:** ethics, law – moral, artificial intelligence, machine learning, lung cancer

## Abstract

**Introduction:**

Artificial intelligence (AI) is increasingly integrating into the healthcare field, particularly in lung cancer care, including screening, diagnosis, treatment, and prognosis. While these applications offer promising advancements, they also raise complex challenges that must be addressed to ensure responsible implementation in clinical practice. This scoping review explores the ethical and legal aspects of AI applications in lung cancer.

**Methods:**

A search was conducted across PubMed, Scopus, Web of Science, Cochrane Library, PROSPERO, OAIster, and CABI. A total of 581 records were initially retrieved, of which 20 met the eligibility criteria and were included in the review. The PRISMA guidelines were followed.

**Results:**

The most frequently reported ethical concern was data privacy. Other recurrent issues included informed consent, no harm to patients, algorithmic bias and fairness, transparency, equity in AI access and use, and trust. The most frequently raised legal concerns were data protection and privacy, although issues relating to cybersecurity, liability, safety and effectiveness, the lack of appropriate regulation, and intellectual property law were also noted. Solutions proposed ranged from technical approaches to calls for regulatory and policy development. However, many studies lacked comprehensive legal analysis, and most included papers originated from high-income countries. This highlights the need for a broader global perspective.

**Discussion:**

This review found that data privacy and protection are the most prominent ethical and legal concerns in AI applications for lung cancer care. Deep Learning (DL) applications, especially in diagnostic imaging, are closely tied to data privacy, lack of transparency, and algorithmic bias. Hybrid and multimodal AI systems raise additional concerns regarding informed consent and the lack of proper regulations. Ethical issues were more frequently addressed than legal ones, with limited consideration for global applicability, particularly in low- and lower middle-income countries. Although technical and policy solutions have been proposed, these remain largely unvalidated and fragmented, with limited real-world feasibility or scalability.

## 1 Introduction

Lung cancer is a significant public health concern, with a global incidence of 2.48 million and mortality of 1.8 million deaths according to GLOBOCAN 2022. It remains the leading cause of cancer-related deaths worldwide (1). Lung cancer is primarily classified into small cell and non-small cell lung cancer (NSCLC), with the latter accounting for approximately 85% of all cases (2). Despite several medical advancements, lung cancer is usually detected at a later stage with over half of patients being diagnosed when curative treatment is no longer an option (3). This late detection coupled with the aggressive nature of lung cancer leading to poor prognosis, with the age-standardized 5-year relative survival rate being between 10–20% in most regions (4). This creates a considerable financial burden on healthcare systems and individuals (5). If left unaddressed, lung cancer is projected to impose the largest global economic burden of all cancers. Tracheal, bronchus, and lung cancers are estimated to account for 15.4% of total costs, amounting to $3.9 trillion by 2050 (6). Therefore, given the significant public health impact of lung cancer, integrating advanced technologies such as AI-driven approaches for early detection and personalized treatment is a clinical imperative to reduce mortality and mitigate the global burden of the disease.

AI refers to the ability of computer systems to perform tasks that are normally done by human reasoning (7). AI consists of Machine Learning (ML) which enables computers to learn from data and modify their decision-making (8). A specialized subset of ML known as DL involves algorithms that process data such as medical images by following a predefined pathway known as an Artificial Neural Network (ANN) (8).

AI technologies are increasingly integrated into the diagnosis and treatment of lung cancer, offering advances in early detection, image interpretation, decision support, and personalized therapy. For instance, AI algorithms now assist in interpreting CT scans for early-stage NSCLC and predicting patient outcomes using radiomics and machine learning models (9). Predictive AI models can accurately stage lung cancer and determine overall survival rates (9, 10). For example, deep learning models such as the neural network developed by Trebeschi et al. can predict one-year overall survival for stage 4 NSCLC by detecting morphological changes across patient follow-up CT scans (11, 12). Similarly, Sybil, a deep learning-based AI algorithm, has produced promising results in predicting the future risk of developing lung cancer from a single Low-Dose CT scan (13). Also, AI models can be trained to provide optimized treatment plans including surgical decision-making, such as surgical risk prediction and assisting in drug selection (9, 14).

Currently, AI has the strongest impact on cancer care in lung cancer imaging diagnostics, where DL algorithms applied to CT scans match human experts in sensitivity (≈82% vs. 81%) while significantly surpassing them in specificity (≈75% vs. 69%) (15). More recently, multi-attention ensemble models have further advanced performance, achieving 98.73% sensitivity and 98.96% specificity in classifying lung nodules from CT images, representing a 35% reduction in error rates compared to previous methods (16).

However, alongside these advances, the use of AI in medicine has raised ethical and legal concerns since its emergence, particularly with regard to patient privacy, bias in algorithms, and accountability for errors (17). Early AI systems like MYCIN in the 1970s highlighted issues of trust and liability despite demonstrating diagnostic potential (18). As modern AI tools are growing more autonomous, scholars emphasize the need for transparent, regulated deployment to ensure equity and safety in healthcare (19).

Some of the ethical concerns include maintaining patient privacy when using large datasets to train models, the interpretability of “black-box” AI systems, and challenges related to informed consent and algorithmic bias of AI models (14, 20, 21).

Legal concerns such as determining liability for AI based technology, data ownership and protection regulations, limit health care workers’ abilities to accurately make health-related decisions (22–24). AI is particularly vulnerable to cyber-attacks which can lead to corrupted data, infected algorithms, or even threats to patient privacy through access to sensitive data (23, 25). These issues underscore the need for responsible AI development and clear ethical and regulatory frameworks as this technology becomes more widely implemented in lung cancer care (22, 26).

However, the ethical and legal challenges in AI-driven cancer research intersect in areas like patient privacy, data ownership, and informed consent, where protecting individuals’ rights is paramount. For example, current data protection regulations, such as the Health Insurance Portability and Accountability Act (HIPAA) in the United States and the General Data Protection Regulation (GDPR) in the European Union, aim to safeguard users’ privacy. They diverge in that ethics often addresses broader questions of fairness, bias, and trustworthiness beyond the law, while legal frameworks focus on compliance with specific regulations and enforceable standards (27).

Given these premises, lung cancer, serves as a critical domain where AI applications are rapidly evolving. The high stakes involved in lung cancer care amplify the consequences of ethical or legal oversights, yet literature discussing these dimensions is dispersed and inconsistently framed across technical, medical, and legal publications.

To date, no comprehensive synthesis has mapped out the breadth of ethical and legal concerns associated with AI in lung cancer care. This scoping review is therefore warranted to systematically explore the existing literature, identify thematic trends, highlight under-researched issues, and outline proposed solutions or regulatory frameworks. In addition, the aim is to answer questions about which categories of ethical and legal concern are most prevalent and which mitigation strategies are being suggested.

## 2 Methods

We selected a scoping review given the novelty of the topic and the heterogeneity of the literature on AI-related ethical and legal concerns in lung cancer. This method was deemed the most appropriate way to map the existing evidence and highlight knowledge gaps. This scoping review was conducted in accordance with the methodological framework developed by Arksey and O’Malley, and guided by the Joanna Briggs Institute (JBI) recommendations, which are widely recognized for ensuring rigor and consistency in evidence synthesis (28, 29). The reporting of the scoping review will follow the PRISMA extension for Scoping Reviews (PRISMA-ScR) checklist (30). The review follows the five-stage process: (1) identifying the research questions, (2) identifying relevant studies, (3) selecting studies, (4) charting the data, and (5) collating, summarizing, and reporting the results.

A protocol outlining the objectives and methodology of this scoping review was registered on the Open Science Framework (OSF) prior to conducting the review. The registration is publicly accessible at https://doi.org/10.17605/OSF.IO/8HUZJ.

### 2.1 Information sources and search strategy

The search strategy combined terms and free-text terms related to lung cancer, artificial intelligence, and ethical/legal concerns. The search query was as follows:

(“lung cancer*” OR “pulmonary cancer*” OR “lung neoplasm*” OR “pulmonary neoplasm*” OR “lung tumo*” OR “lung nodule*” OR “pulmonary nodule*”) AND (“artificial intelligence” OR “machine learning” OR “deep learning” OR “computer reasoning” OR “computational intelligence” OR “machine intelligence” OR “neural network*” OR algorithm* OR robotics) AND (ethic* OR moral* OR bioethic* OR jurisprudence OR litigat* OR legal* OR policy OR policies OR law*).

A comprehensive literature search was performed using the following electronic databases: PubMed, Scopus, Web of Science, Cochrane Library, and PROSPERO. To capture the full scope of the literature and ensure comprehensive coverage of the field, we included grey literature by searching OAIster and CABI. The search was conducted without restrictions on language or publication date. Search strategies were adapted for each database as needed. Additionally, a snowball search was conducted by screening the reference lists of the articles included. The full search strategy, including the search queries used in each database, is provided in Supplementary file S1.

### 2.2 Eligibility criteria

The eligibility criteria were developed based on the Population–Exposure–Outcome (PEO) framework:

- Population (P): Patients with lung cancer at any stage, including those undergoing screening, diagnosis, treatment, or prognostic assessment.
- Exposure (E): Use of AI technologies in lung cancer care, including imaging analysis, predictive modeling, clinical decision-support systems, treatment planning, and prognostic assessment.
- Outcome (O): Ethical and legal issues arising from the use of AI in lung cancer care, as well as proposed solutions and mitigation strategies.

Studies were included if the full text was available and written in English. All types of publications, including original research articles, reviews, conference papers or proceedings, grey literature, editorials, opinions, letters, and commentaries, were included, while study protocols were excluded. The content of the publication needed to be relevant to lung cancer, either focusing directly on its diagnosis, treatment, screening, or prognosis, or mentioning lung cancer within broader discussions of multiple diseases. Additionally, studies had to involve the use of artificial intelligence techniques, such as machine learning or deep learning, in relation to lung cancer. Articles that mentioned any ethical or legal discussions were included if they focused on lung cancer or, in the case of multi-disease discussions, made explicit reference to lung cancer within the ethical or legal context.

The categories used to classify ethical and legal concerns were adopted from the study by Gerke et al. (23).

The ethical concerns were categorized as follows: informed consent to use, safety and transparency, algorithmic bias and fairness, and data privacy. The legal concerns were categorized as follows: safety and effectiveness, liability, data protection and privacy, cybersecurity, intellectual property law. Additional ethical and legal concerns not covered by these categories can be included as “other.”

### 2.3 Selection of sources of evidence

The identified records were imported from each database into Endnote. Then, they were imported into Covidence for duplication removal and screening. Two independent reviewers conducted the initial data extraction (GC, NA). Any discrepancies were addressed through weekly consensus meetings (lasting approximately 1.5 h each). When consensus could not be reached, a third reviewer (OV) was consulted to adjudicate and provide a final decision.

### 2.4 Data charting process and data items

A data extraction form was developed and pilot-tested within Covidence. Two reviewers (GC, NA) independently charted the data, with discrepancies discussed and resolved by consensus with the help of a third party (OV). The following data items were extracted from each included study: publication ID, title, lead author, year of publication, country of affiliation, source type, aim of the publication, type of lung cancer discussed, AI-based technology addressed, application of AI technology in lung cancer care, ethical concerns, legal concerns, and suggested solutions.

### 2.5 Synthesis of results

Extracted data were collated and summarized in tabular form to provide an overview of the characteristics and scope of the included literature. A descriptive synthesis was conducted to map the ethical and legal concerns raised in relation to the use of AI in lung cancer screening, diagnosis, treatment, and prognosis including the types of technologies used, geographic distribution of studies, and recurring themes in ethical and legal context.

## 3 Results

### 3.1 Selection of sources of evidence

**Figure 1:**
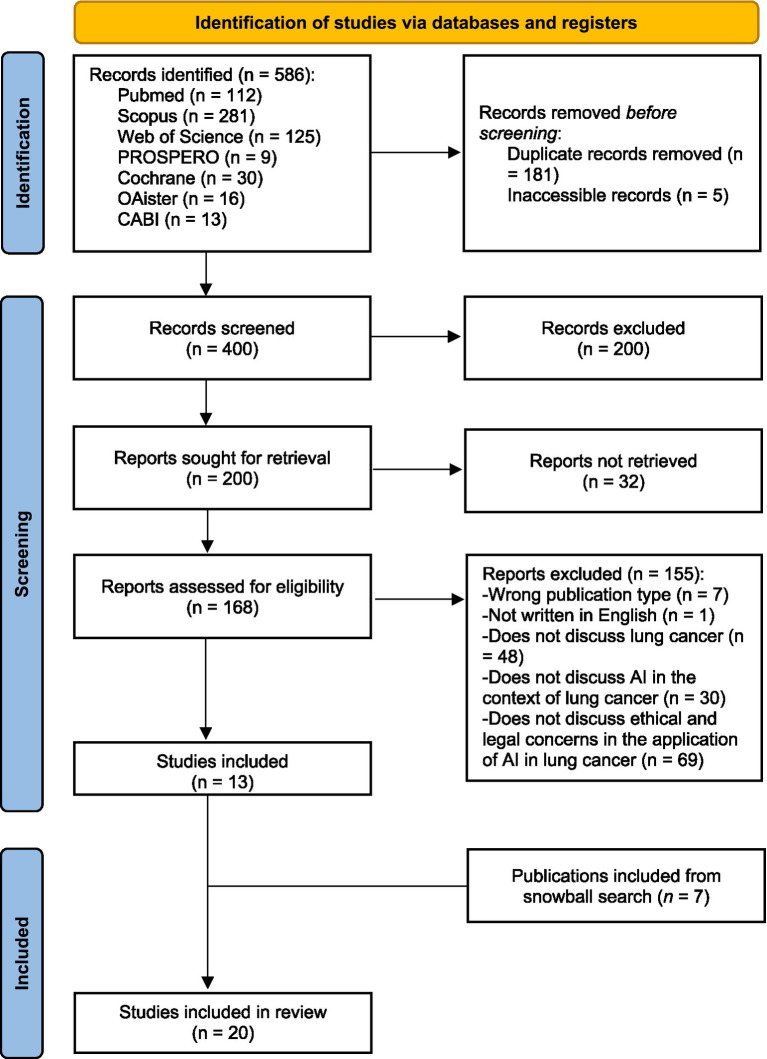
Preferred reporting items for systematic reviews and meta-analyses (PRISMA 2020) flowchart.

After applying the search strategy across all databases, 581 records were retrieved. Following duplicate removal and initial screening, 400 articles were reviewed at the title and abstract level. Of these, 200 were excluded, and 200 proceeded to full-text screening. Among the remaining 200 records, 32 did not have accessible full texts. A total of 168 articles were assessed for eligibility criteria. Of these, 155 were excluded: 7 publications were excluded due to being the wrong type (protocols), 1 publication was in a language other than English, 48 did not discuss lung cancer, 30 did not discuss AI in lung cancer, and 69 did not include any ethical or legal discussion concerning the application of AI in lung cancer. After further exclusions based on eligibility criteria, a total of 13 studies were included. An additional 7 relevant records were identified through snowball searching, bringing the total number of included publications to 20 (Figure 1).

### 3.2 Synthesis of results

#### 3.2.1 General characteristics of relevant studies

Our search identified studies published between 1996 and 2024, the year with the most publications was in 2021 [7 publications; (31–37)]. The distribution of publications based on the first author’s affiliations shows that the most frequently affiliated country is China [4 publications; (35, 36, 38, 39)], followed by India [3 publications; (40–42)], then 2 publications each for Italy (33, 34), France (43, 44), United States (45, 46), and Australia (31, 32), while the remaining countries (the United Kingdom, Greece, Norway, Germany, and Canada) had one publication each (37, 47–50). Out of the 20 studies, 18 were journal articles, and 2 were conference proceedings publications (41, 46) (Table 1).

**Table 1:** Main characteristics of the included studies.

Title	Author (first)	Year of publication	Country of affiliation	Source type	Aim of publication
Demographic bias in misdiagnosis by computational pathology models (45)	Anurag Vaidya	2024	United States	Journal article	To assess the performance of state-of-the-art computational pathology approaches across different demographic subgroups, including racial and income groups, for binary classification of subtypes of breast and lung carcinomas and for predicting mutations in gliomas
An enhanced multimodal fusion deep learning neural network for lung cancer classification (40)	Sangeetha S. K. B.	2023	India	Journal article	To gather diverse datasets, including medical images, genomic data, and clinical records, and to assess their suitability and to design a deep neural network architecture for multimodal fusion
An Integration of blockchain and AI for secure data sharing and detection of CT images for the hospitals (38)	Rajesh Kumar	2020	China	Journal article	To propose a novel method that combines various deep learning models over a blockchain to improve lung cancer detection and self-learning through a decentralized network
Machine learning-based classification of lung cancer types from radiological images (41)	Amit Joshi	2023	India	Conference proceeding paper	To create a machine learning-powered classification method for identifying various forms of lung cancer using radiological images.
Deep mining generation of lung cancer malignancy models from chest X-ray images (31)	Michael Horry	2021	Australia	Journal article	To present a novel framework that automatically generates interpretable models for the stratification of lung cancer CXR^a^ images into benign and malignant samples
Implementation of the Australian computer-assisted theragnostics (AusCAT) network for radiation oncology dataآ extraction, reporting and distributed learning (32)	Matthew Field	2021	Australia	Journal article	To demonstrate the feasibility of automatically extracting, de-identifying, and standardizing datasets; assessing data availability and quality for this patient cohort; securely and efficiently developing and validating machine learning–based outcome-prediction models; and to validate an overall survival model by externally evaluating its performance in patients with unresectable Stage I–III NSCLC treated with radiotherapy
Federated learning of lung nodule detection based on dual mechanism differential privacy protection (39)	Kefeng Fan	2024	China	Journal article	To propose federated learning for lung nodule detection, which collaboratively builds shared machine learning models without exposing local datasets.
Artificial intelligence in thoracic surgery: past, present, perspective and limits (43)	Harry Etienne	2020	France	Journal article	To review the applications of AI to thoracic surgery, highlight the outlook in robotic surgery, and discuss the limits, ethical and legislative issues of widespread application of AI in thoracic surgery, in the European Union
Combining liquid biopsy and radiomics for personalized treatment of lung cancer patients. State of the art and new perspectives (33)	Federico Cucchiara	2021	Italy	Journal article	To improve precision medicine in oncology, particularly for lung cancer cases
Design-based approach to ethics in computer-aided diagnosis (46)	Jeff R. Collmann	1996	United States	Conference proceeding paper	To resolve central technical questions in designing clinically functional CADx^b^ systems for lung cancer and breast cancer diagnosis.
Artificial intelligence in thoracic surgery: a narrative review (34)	Valentina Bellini	2021	Italy	Journal article	To review the current applications of artificial intelligence in thoracic surgery, from diagnosis and pulmonary disease management, to preoperative risk-assessment, surgical planning, and outcomes prediction.
Secret learning for lung cancer diagnosis-a study with homomorphic encryption, texture analysis and deep learning (42)	Subhrangshu Adhikary	2023	India	Journal article	To propose a method for applying homomorphic encryption to CT scan images of various types of lung cancer; to extract texture information that enables classification of homomorphically encrypted images; and to apply deep learning for automated classification of lung cancer on encrypted data
The future of artificial intelligence in thoracic surgery for non-small cell lung cancer treatment a narrative review (47)	Namariq Abbaker	2024	United Kingdom	Journal article	To explore the current state of AI integration in thoracic surgery for NSCLC treatment.
The application of artificial intelligence in lung cancer: a narrative review (35)	Huixian Zhang	2021	China	Journal article	To summarize the progress made by AI technology in early screening based medical imaging, pathological diagnosis, genomics inspection, prognostic evaluation, and individual treatment of lung cancer.
A systematic review and meta-analysis of diagnostic performance and physicians’ perceptions of artificial intelligence (AI)-assisted CT diagnostic technology for the classification of pulmonary nodules (36)	Guo Huang	2021	China	Journal article	To systematically review the diagnostic performance of AI-assisted CT technology in classifying pulmonary nodules as benign or malignant; and to analyze physicians’ perceptions of its potential benefits and risks, as well as their attitudes toward its clinical application
Deep learning for lung cancer diagnosis, prognosis and prediction using histological and cytological images: a systematic review (48)	Athena Davri	2023	Greece	Journal article	To provide an overview of the current advances in DL-based methods on lung cancer by using histological and cytological images
Artificial intelligence: a critical review of applications for lung nodule and lung cancer (44)	Constance de Margerie-Mellon	2022	France	Journal article	To review and discuss the current and future applications of AI in the elective field of lung nodule and lung cancer.
Pulmonary nodule classification in lung cancer from 3D thoracic CT scans using fastai and MONAI (37)	Satheshkumar Kaliyugarasan	2021	Norway	Journal article	To classify pulmonary nodules as malignant or benign in the context of lung cancer.
Deep learning for the classification of small-cell and non-small-cell lung cancer (49)	Mark Kriegsmann	2020	Germany	Journal article	To classify the most common lung cancer subtypes and develop quality control measures to objectively identify cases requiring further evaluation
Role of artificial intelligence in the care of patients with non-small cell lung cancer (50)	Mohamad Rabbani	2018	Canada	Journal article	To review machine learning applications developed for the detection and treatment of NSCLC, as well as the current challenges facing clinical adoption.

a CXR: Chest X-ray.

b CADx: Computer-Aided Diagnosis.

#### 3.2.2 Overview of the applications of AI in lung cancer

The reviewed studies demonstrate diverse applications of AI for different aspects of lung cancer care. The use of AI was classified into 4 categories: screening, diagnosis, treatment, and prognosis. In the context of screening, AI was employed to detect pulmonary nodules on chest radiographs (39, 43, 50), and to identify target sites and detect lung nodules in images using CADe (Computer-Aided Detection) systems (35, 44) (Table 2).

**Table 2:** Overview of the AI algorithm and the ethical and legal concerns in the included studies.

Author (year)	AI technology	Application of AI technology	Categories of AI applications	Ethical principles related to the concern	Legal principles related to the concern
Vaidya et al. (2024) (45)	DL	To distinguish between lung adenocarcinoma and lung squamous cell carcinoma	Diagnosis	Algorithmic fairness and biases	-
Sangeetha et al. (2023) (40)	Multimodal Fusion Deep Neural Network	To integrate and process data from diverse sources, including medical images, genomic data, and clinical records; and to perform binary classification of lung cancer cases as cancerous or non-cancerous.	Diagnosis	Data privacy, informed consent to use, safety and transparency	Data protection and privacy, liability
Kumar et al. (2020) (38)	DL (RCNN^a^)	To detect lung cancer in radiological images and estimate the region of interest in the CT images.	Diagnosis	Data privacy	Data protection and privacy
Joshi et al. (2023) (41)	CNN^b^, SVM^c^	To identify various forms of lung cancer using radiological images.	Diagnosis	Data privacy, informed consent to use	-
Horry et al. (2021) (31)	DL, DT^d^	To stratify lung cancer patient CXR images from an independent dataset into benign/malignant categories.	Diagnosis	Data privacy	-
Field et al. (2021) (32)	Distributed learning approach, SVM	To extract and report on oncology data and validate an overall survival model in patients with unresectable Stage I–III NSCLC treated with radiotherapy	Prognosis	Data Privacy	Data protection and privacy
Fan et al. 2024 (39)	Federated learning algorithm, CNN	To solve the problem of small size and fragmentation of medical data, without exposing local private data by proposing federated learning for lung nodule detection	Screening	Data Privacy	Data protection and privacy, cybersecurity
Etienne et al. (2020) (43)	ML, DL (CNN)	To distinguish between benign and malignant nodules, detect nodules on chest radiographs, differentiate lung adenocarcinoma from squamous cell carcinoma using pathology slides, predict gene mutations, support decision-making for surgery patients by evaluating individual surgical risk factors, and adapt decision making individually, support Robotic-Assisted Surgery.	Screening, Diagnosis, Treatment, Prognosis	Data privacy, no harm to patients	Data protection and privacy, liability, safety and effectiveness
Cucchiara et al. (2021) (33)	ML, DL	To link patients’ clinical data with tumor molecular profiles and imaging characteristics; and to implement radiomics and liquid biopsy for integrated analysis	Diagnosis, Treatment, prognosis	-	Data protection and privacy, intellectual property law
Collmann et al. (1996) (46)	ANN	To distinguish true positives from false positives in the diagnosis of lung cancer	Diagnosis	No harm to patients	-
Bellini et al. (2021) (34)	DL(CNN), ML (XGBOOST, SVM, random forest, DT)	To diagnose and detect pulmonary nodules using CADx; to predict the risk of major complications and mortality following lung resection; to reduce hospital stay duration and postoperative complications through the use of surgical robotics; to distinguish between lung cancer types in pathological analysis; and to predict the risk of lung adenocarcinoma recurrence.	Diagnosis, Prognosis, Treatment	Informed consent to use, equity in access and use	Data protection and privacy
Adhikary et al. (2023) (42)	Deep neural network	To classify CT scanned images of three types of lung cancer	Diagnosis	Data privacy	-
Abbaker et al. (2024) (47)	DL (CNN, RNN, ANN)	To classify challenging cytological slide images from lung samples and predict lung cancer–related IHC phenotypes; to classify pulmonary nodules on CT scans and assist surgeons by identifying anatomical structures and aiding decision-making; to reduce delays in post-surgery diagnoses and estimate postoperative prognosis; to predict therapy responses, assess surgical risks, and support cancer staging; to predict genetic mutations such as ALK rearrangements and EGFR mutations; to estimate cardiorespiratory morbidity and postoperative outcomes; and to provide personalized drug treatment recommendations guiding targeted therapy selection and surgical planning.	Diagnosis, Treatment, Prognosis	Informed consent to use, safety and transparency, Algorithmic fairness and biases, Data Privacy, trust	Accountability
Zhang et al. (2021) (35)	DL (CNN), ML (SVM, DT), CDSS	To identify target sites in clinical images to assist imaging inspections using CADe and CADx systems; to analyze ambiguous morphology in histopathological images to support diagnosis; to detect minimal biomarker presence in liquid biopsy; to support clinical decision-making using a CDSS; to enhance surgical precision and reduce invasiveness via RATS; and to plan personalized treatment by regulating irradiation time, dose rate, and imaging in radiotherapy	Screening, Diagnosis, Treatment, Prognosis	Data privacy	Lack of regulation
Huang et al. (2021) (36)	SVMs, CNN, ANN, BN, Fuzzy C-means	To classify pulmonary nodules as benign or malignant	Diagnosis	Data privacy, No harm to patients	Data protection and privacy
Davri et al. (2023) (48)	ML, DL	To use histological data to assist in lung cancer diagnosis; to support prognosis estimation and mutational status assessment; to aid cytological interpretation; and to evaluate programmed cell death ligand 1 expression	Diagnosis, Prognosis	Data privacy	-
De Margerie-Mellon et al. (2022) (44)	CNN	To detect lung nodules using DL-based CADe algorithms in CXR and CT scans; to distinguish benign from malignant nodules using CADx; to assist in lung nodule segmentation; to predict mutations; to stratify patients into low- and high-mortality risk groups after radiotherapy and surgery; and to predict survival and cancer-specific outcomes	Screening, diagnosis, treatment, prognosis	Liability,	Liability
Kaliyugarasan et al. (2021) (37)	CNN	To classify pulmonary nodules as malignant or benign	Diagnosis	Safety and transparency	-
Kriegsmann et al. (2020) (49)	CNN	To differentiate the most common lung cancer subtypes	Diagnosis	No harm to patients	-
Rabbani et al. (2018) (50)	ML (DT, SVM), ANN	To detect solid, nonsolid, and cavitary nodules; to discriminate benign from malignant tumors; to identify genetic subtypes of NSCLC; to select the optimal radiation beam angle through dose–volume histogram predictions; to predict cancer subtype, tumor growth, metastatic potential, and patient survival; and to improve patient selection and prognostic models for predicting early mortality or treatment failure	Screening, Diagnosis, Prognosis, Treatment	Data privacy, data ownership	Lack of proper regulation, data protection and privacy, cybersecurity risks

a RCNN, Region-based Convolutional Neural Network.

b CNN, Convolutional Neural Network.

c SVM, Support Vector Machine.

d DT, Decision Tree.

Beyond detection, AI plays a role in lung cancer diagnosis, which was the most common application. The majority of publications reported the use of AI algorithms or AI-based systems to classify pulmonary nodules as malignant or benign, or to distinguish between lung cancer subtypes (31, 34, 36, 37, 40–43, 45–47, 49, 50). Additionally, AI tools were used to support lung cancer diagnosis using histological data. Applications included differentiating lung cancer types in pathology, classifying challenging cytological slide images, and analyzing ambiguous morphology in histopathological images from lung cancer biopsies (34, 35, 47, 48).

AI has been used to support various aspects of lung cancer treatment. Etienne et al. demonstrated that AI can assist in surgical procedures and support decision-making, including the use of robotics (43). Bellini et al. reported that AI-assisted surgery can reduce hospital stay and postoperative complications (34). In another study, it was shown that AI could contribute to personalized drug treatment recommendations and to guide targeted therapy selection and surgical planning (47). Similarly, Zhang et al. applied AI to enhance surgical precision and reduce invasiveness via RATS (Robot-Assisted Thoracic Surgery), as well as to plan personalized treatment and regulate irradiation time, dose rate, and imaging in radiotherapy (35). Rabbani et al. further advanced radiation therapy by using AI to predict dose-volume histograms and select the optimal angle for radiation (50). Additionally, Cucchiara et al. explored the integration of AI with radiomics and liquid biopsy for therapeutic purposes (33).

AI technologies were employed in several studies to support lung cancer prognosis. One study reported that AI was used to assist surgical decision-making by evaluating individual risk factors and enabling personalized clinical decisions (43). In another study, AI was applied to predict the risk of major complications and mortality after lung resection, as well as the risk of lung adenocarcinoma recurrence (34). In addition, Abbaker et al. focused on estimating postoperative prognosis, predicting therapy responses, assessing surgical risk, and forecasting cardio-respiratory morbidity and postoperative outcomes (47). Histological data were also used to support prognosis (48). AI was further used to stratify patients by mortality risk following radiotherapy and surgery, and to predict survival and cancer-specific outcomes (44). Prognostic models were also developed for early mortality and treatment failure (50).

#### 3.2.3 Overview of ethical and legal concerns identified

The analysis of 20 included studies revealed consistent ethical and legal challenges associated with AI applications in lung cancer (Table 2). Ethically, the most prominent concerns centered on data privacy [13 publications; (31, 32, 35, 36, 38–43, 47, 48, 50)], particularly in contexts involving sensitive imaging or genomic data, and the need for robust informed consent mechanisms. The principle of non-maleficence or causing no harm to patients emerged as the second critical issue [4 publications; (36, 43, 46, 49)]. Studies highlighted risks to patient lives if AI systems fail to distinguish true from false-positive lung lesions, or provide inappropriate or inaccurate risk assessments, treatment recommendations, or diagnoses. Similarly, informed consent-related concerns [4 publications; (34, 40, 41, 47)] was identified as essential to upholding patient autonomy and ensuring comprehension in diagnostic and surgical decision-making. Furthermore, safety and transparency deficits in “black-box” deep learning models [3 publications; (37, 40, 47)] underscored the need for interpretable decision-making processes to ensure model reliability.

The principle of Algorithmic fairness and bias was emphasized in two studies [2 publications; (45, 47)]. They noted that biased or under-representative training datasets could lead to unfair outcomes.

Equity in access and use [1 publication; (34)] emerged as a critical concern, whether in access to AI technologies or disparities in digital literacy among users. Addressing these issues is essential to ensure equitable demographic distribution of AI tools. Moreover, trust in AI systems [1 publication; (47)] was identified as a challenge, with the opaque nature of AI algorithms cited as a barrier to enhancing trustworthiness. Finally, liability within ethical frameworks [1 publication; (44)] was noted, raising questions about the extent of accountability for AI-driven decisions.

Legally, data protection and privacy [9 publications; (32–34, 36, 38–40, 43, 50)] dominated discussions, with studies highlighting compliance challenges under regulations such as GDPR or HIPAA. Liability ambiguities [3 publications; (40, 43, 44)] emerged, particularly surrounding responsibility for errors generated by AI tools. Cybersecurity concerns [2 publications; (39, 50)] were raised regarding potential hacking threats to datasets used in algorithms. Notably, only two studies comprehensively addressed the lack of proper regulation and legislation governing AI integration in lung cancer care (35, 50). A single study highlighted safety and effectiveness concerns, emphasizing that AI tools should be evaluated to meet legal requirements (43). Another study discussed intellectual property law and the importance of addressing regulatory aspects related to AI algorithm ownership (33). Finally, accountability was mentioned in one study as a key consideration (47).

#### 3.2.4 Overview of the solutions

A total of 20 studies were reviewed to identify ethical and legal considerations in the use of AI for lung cancer diagnosis and treatment. 15 out of 20 suggested solutions for the ethical concerns presented (Table 3).

**Table 3:** The solutions proposed for the ethical/legal concern in the included publications.

Author	Ethical/legal principle related to concern	Suggested solution
Vaidya et al. (45)	Algorithmic fairness and biases	Using a bias mitigation strategy like the importance weighting that often-reduced disparity, but at the cost of performance.
SKB et al. (40)	Data privacy	Using robust encryption, anonymization techniques, and access controls in the patient data
	Liability	Establishing a clear guideline for the development and deployment of AI in healthcare
Kumar et al. (38)	Data privacy, Data protection and privacy	Using the blockchain-based which is a novel multi-model method that combines deep learning and blockchain technology
Horry et al. (31)	Data privacy	Utilizing state-of-the-art signal-to-noise improvement techniques applied to the CXR pre-processing pipeline, customization of the deep learning feature extraction algorithm to include wavelet filtering, followed by reference implementation in a federated deep learning framework
Field et al. (32)	Data privacy, Data protection and privacy	Using the distributed learning approach to model validation and development using the AusCAT platform
Fan et al. (39)	Data privacy, Data protection and privacy	Using dual mechanism differential privacy applied to federated learning, which improves the accuracy of the model under the premise that the patient’s personal privacy is guaranteed
Etienne et al. (43)	Liability	Considering a specific legal status for robots as “electronic persons”, responsible for making good any damage they may cause
Cucchiara et al. (33)	Data protection and privacy	Regulating large reserves of linked information through proper policies. Strong authentication methods and traceability. Linking and coordinating of medical records
Bellini et al. (34)	Data protection and privacy	Developing specific guidelines for the protection of personal and extremely sensitive information.
Adhikary et al. (42)	Data privacy	Utilizing homomorphic encryption has been utilized in this paper to preserve the privacy of the patient by encrypting the CT-Scan images on which computations can be performed
Abbaker et al. (47)	Algorithmic fairness and biases	Creating a robust regulatory framework for AI in healthcare
Huang et al. (36)	Data protection and privacy	Following measures to protect patient privacy and sensitive health information during the collection
Davri et al. (48)	Data privacy	Creating a regulatory framework to protect patient’s rights and ensure the security of sensitive medical data and confidentiality
Kaliyugarasan et al. (37)	Safety and transparency	Gaining some explainability for image classification models by using CAM and Grad-CAM
Rabbani et al. (50)	Data privacy	Using strong user authentication methods to take into account the high-security constraints. Harmonizing the regulatory framework to ensuring the personal data protection and compliance with legal requirements.
	Data ownership	Creating policies that properly regulate the large federated data reserves.
Kriegsmann et al. (49)	No harm to patients	Applying CNN for tumor classification must always be conducted under the supervision of a pathologist to avoid misdiagnosis and potentially harmful consequences for patients.

##### 3.2.4.1 Ethical solutions

Several studies addressed key ethical concerns, with data privacy being the most commonly cited issue for which solutions were suggested (31, 32, 38–40, 42, 48, 50). Davri et al. (48) proposed the creation of a regulatory framework to ensure data security and confidentiality. Additionally, Rabbani et al. (50) emphasized the importance of a legal framework to protect personal data. Another solution proposed by Rabbani et al. involves the use of strong authentication methods. Other papers also proposed technical solutions, recommending the use of encryption (40, 42). Kumar et al. (38) suggested using blockchain in combination with DL, while others advocated decentralized methods, such as distributed learning or federated deep learning (31, 32).

To avoid harming patients, Kriegsmann et al. recommend that AI algorithms be supervised by humans to prevent misdiagnosis (49).

One study suggested that class activation maps (CAM) and gradient-weighted CAM (Grad-CAM) can improve model explainability, addressing safety and transparency concerns in deep learning (37).

Concerning algorithmic fairness and bias, Vaidya et al. (45) suggested a bias mitigation strategy, while Abbaker et al. (47) suggested a legal framework for AI in healthcare.

The study by Rabbani et al. (50) discusses data ownership as an ethical concern that should be addressed through appropriate policy regulation.

##### 3.2.4.2 Legal solutions

As ethical and legal concerns often overlap, some of the solutions proposed for data protection and privacy in the reviewed publications were intended to address the same ethical concern: data privacy. Clearly separating the two domains was difficult.

Many ethical concerns have corresponding legal solutions. These include the use of a blockchain-based data sharing method (38) and distributed learning approaches (32, 39). Furthermore, Cucchiara et al. (33) and Bellini et al. (34) discussed data protection regulations in their studies, recommending compliance with robust legal frameworks. Also, Huang et al., (36) emphasized the importance of a regulatory framework to safeguard sensitive medical data and ensure confidentiality.

Two studies proposed legal solutions involving the creation of guidelines and the establishment of a legal framework to address liability issues (40, 43).

## 4 Discussion

This scoping review systematically identified the predominant ethical and legal concerns associated with AI applications in lung cancer care, as well as the proposed solutions to address these concerns.

### 4.1 Overall findings

Of the identified ethical and legal concerns, issues related to data privacy and data protection were found to be the most significant. This finding aligns with the work of Cartolovni et al., whose study on AI-based medical decision-support tools similarly identified privacy considerations as a major ethical and legal challenge (51).

The use of big data for training and validating AI algorithms is fundamentally important (52), yet it inevitably raises significant privacy concerns, making robust data protection measures essential (53). Several established frameworks address these issues, including the GDPR in May 2018 in Europe, the HIPAA in the United States for health data protection, and the Global Initiative on Ethics of Autonomous and Intelligent Systems. Beyond regulatory compliance, the ethical necessity to protect privacy has actively driven technological innovation, leading to the development of AI models with privacy preservation mechanisms such as federated learning (31, 39). Furthermore, several studies (*n* = 8), including those by Joshi et al. (41) and Horry et al. (31), have recognized ethical concerns such as data privacy and informed consent, but did not take into account any legal concerns. This reflects a trend where the focus is more on ethical issues than legal ones, not just in lung cancer but also across the broader healthcare field (54).

The predominance of studies from high-income and upper-middle-income countries introduces an important limitation for the generalizability of our findings. As most of the included studies originate from China, Italy, France, Australia, and the U.S., there is a lack of information on how AI will function ethically and legally in low- and lower middle-income countries (31–36, 38, 39, 43–46). Such an absence of studies raises concerns about global equity. It also limits our understanding of how to implement AI in contexts where healthcare infrastructures, regulatory environments, and cultural perspectives on ethics may differ substantially. Future research should critically examine these disparities and include studies from diverse regions to ensure that AI applications in lung cancer are equitable, context-sensitive, and globally relevant.

### 4.2 Patterns between AI types and ethical/legal concerns

Specific relationships between AI types, application areas, and the nature of ethical/legal concerns can be observed, although the evidence remains uneven. Diagnostic DL applications, particularly in imaging, are most often associated with risks of data privacy, a lack of transparency/interpretability, and algorithmic bias. This reflects their “black box” nature and their reliance on large datasets (37, 38, 40, 42, 45, 47). Hybrid or multimodal AI systems integrate clinical records, genomic data, and imaging. They raise compounded challenges, including data privacy, informed consent, and a lack of regulatory oversight (31, 32, 35, 41, 43, 48, 50).

The type of AI application directly influences the nature of the ethical and legal concerns it raises. Diagnostic DL models tend to prioritize issues of privacy and transparency, while hybrid approaches used in all lung care areas, frequently highlight gaps in existing regulations. Yet, most studies address these concerns in general terms, without explicitly linking them to the architecture or operational context of the AI systems involved.

### 4.3 Validity and practicality of proposed solutions

Although technical solutions such as homomorphic encryption (42), federated learning (31, 39), and Grad-CAM explainability (37) show promising results. However, they are primarily reported in experimental or small-scale contexts. Their deployment on a clinical scale is rarely validated. Blockchain for privacy (38) and bias mitigation algorithms (45) have also been proposed, but trade-offs—such as reduced model performance or higher computational demands—are rarely assessed. Only one study (45) acknowledged that bias mitigation reduced performance.

Policy proposals, such as labeling robots as “electronic persons” (43), have been described as legally ambiguous and inconsistent with current jurisprudence, potentially undermining real-world applicability. Legal recommendations, like clearer data ownership laws (50) lack jurisdiction-specific detail, particularly regarding interoperability between regulatory frameworks like GDPR and HIPAA.

Taken together, the proposed solutions can be synthesized into three broad categories: technical safeguards (e.g., encryption, federated learning, blockchain, explainability tools), legal structures (e.g., liability frameworks, data ownership regulations), and policy guidelines (e.g., international standards or governance frameworks). While these approaches highlight possible pathways forward, they are often presented in isolation and rarely assessed for feasibility, scalability, or readiness for clinical use, a limitation also noted in previous reviews of AI governance proposals (51). Technical measures may enhance privacy but reduce performance; legal structures can improve accountability but face jurisdiction barriers; and policy guidelines often remain aspirational. Consequently, most proposals remain abstract. These trade-offs underscore the need for future research that critically examines not only the conceptual merit of these proposals but also their operational viability in diverse healthcare settings.

## 5 Conclusion

This review surfaces a vital concern: while ethical and legal issues widely acknowledged, the depth of analysis often remains surface-level, lacking in specificity and operational grounding. Ethical concerns are explored more than legal ones, and the mapping between AI type, clinical application, ethical and legal implications and actionable solutions is still underdeveloped. A meaningful step forward would be to develop context-aware, AI-type-specific governance frameworks that are technically feasible, legally binding, and globally inclusive, a need not currently addressed by the literature.
