# Supplementary material for: Ethical and legal concerns in artificial intelligence applications for the diagnosis and treatment of lung cancer: a scoping review
Source: Front Public Health. 2025 Oct 14;13:1663298. doi: 10.3389/fpubh.2025.1663298 (PMC12558840; doi:10.3389/fpubh.2025.1663298)
Supplement: Supplementary file 1 [file Table_1.docx]

**Supplementary File S1.** Customized search strategies utilized for each database, along with the number of studies retrieved.

| Database | Search terms | Hits |
| --- | --- | --- |
| Pubmed | ("lung neoplasms"[MeSH Terms] OR ("lung cancer*"[Title/Abstract] OR "pulmonary cancer*"[Title/Abstract] OR "lung neoplasm*"[Title/Abstract] OR "pulmonary neoplasm*"[Title/Abstract] OR "lung tumo*"[Title/Abstract] OR "lung nodule*"[Title/Abstract] OR "pulmonary nodule*"[Title/Abstract])) AND ("artificial intelligence"[MeSH Terms] OR ("artificial intelligence"[Title/Abstract] OR "machine learning"[Title/Abstract] OR "deep learning"[Title/Abstract] OR "computer reasoning"[Title/Abstract] OR "computational intelligence"[Title/Abstract] OR "machine intelligence"[Title/Abstract] OR "neural network*"[Title/Abstract] OR "algorithm*"[Title/Abstract] OR "robotics"[Title/Abstract])) AND ("ethics"[MeSH Terms] OR "bioethics"[MeSH Terms] OR "morals"[MeSH Terms] OR "Jurisprudence"[MeSH Terms] OR "Legislation as Topic"[MeSH Terms] OR "ethic*"[Title/Abstract] OR "moral*"[Title/Abstract] OR "bioethic*"[Title/Abstract] OR "Jurisprudence"[Title/Abstract] OR "litigat*"[Title/Abstract] OR "legal*"[Title/Abstract] OR "policy"[Title/Abstract] OR "policies"[Title/Abstract] OR "law"[Title/Abstract]) | 112 |
| Scopus | ( TITLE-ABS-KEY ( "lung cancer*" OR "pulmonary cancer*" OR "lung neoplasm*" OR "pulmonary neoplasm*" OR "lung tumo*" OR "lung nodule*" OR "pulmonary nodule*" ) ) AND ( TITLE-ABS-KEY ( "artificial intelligence" OR "machine learning" OR "deep learning" OR "computer reasoning" OR "computational intelligence" OR "machine intelligence" OR "neural network*" OR algorithm* OR robotics ) ) AND ( TITLE-ABS-KEY ( ethic* OR moral* OR bioethic* OR jurisprudence OR litigat* OR legal* OR policy OR policies OR law* ) ) | 281 |
| Web of science | 1: TS=("lung cancer*" OR "pulmonary cancer*" OR "lung neoplasm*" OR "pulmonary neoplasm*" OR "lung tumo*" OR "lung nodule*" OR "pulmonary nodule*" )  2: TS=("artificial intelligence" OR "machine learning" OR "deep learning" OR "computer reasoning" OR "computational intelligence" OR "machine intelligence" OR "neural network*" OR algorithm* OR robotics )  3: TS=(ethic* OR moral* OR bioethic* OR jurisprudence OR litigat* OR legal* OR policy OR policies OR law* )  4: #1 AND #2 AND #3 | 125 |
| Prospero | #1 "lung cancer*" OR "pulmonary cancer*" OR "lung neoplasm*" OR "pulmonary neoplasm*" OR "lung tumo*" OR "lung nodule*" OR "pulmonary nodule*"  #2 MeSH DESCRIPTOR lung neoplasms EXPLODE ALL TREES  #3 "artificial intelligence" OR "machine learning" OR "deep learning" OR "computer reasoning" OR "computational intelligence" OR "machine intelligence" OR "neural network*" OR algorithm* OR robotics  #4 MeSH DESCRIPTOR artificial intelligence EXPLODE ALL TREES  #5 ethic* OR moral* OR bioethic* OR jurisprudence OR litigat* OR legal* OR policy OR policies OR law*  #6 MeSH DESCRIPTOR ethics EXPLODE ALL TREES  #7 MeSH DESCRIPTOR bioethics EXPLODE ALL TREES  #8 MeSH DESCRIPTOR morals EXPLODE ALL TREES  #9 MeSH DESCRIPTOR jurisprudence EXPLODE ALL TREES  #10 MeSH DESCRIPTOR Legislation as Topic EXPLODE ALL TREES  #11 #1 OR #2  #12 #3 OR #4  #13 #5 OR #6 OR #7 OR #8 OR #9 OR #10  #14 #11 AND #12 AND #13 | 9 |
| Cochrane | #1 ((lung NEXT cancer*) OR (pulmonary NEXT cancer*) OR (lung NEXT neoplasm*) OR (pulmonary NEXT neoplasm*) OR (lung NEXT tumo*) OR (lung NEXT nodule*) OR (pulmonary NEXT nodule*)):ti,ab,kw (Word variations have been searched)  #2 MeSH descriptor: [Lung Neoplasms] explode all trees  #3 #1 OR #2  #4 ("artificial intelligence" OR "machine learning" OR "deep learning" OR "computer reasoning" OR "computational intelligence" OR "machine Intelligence" OR (neural NEXT network*) OR algorithm* OR Robotics):ti,ab,kw (Word variations have been searched)  #5 MeSH descriptor: [Artificial Intelligence] explode all trees  #6 #4 OR #5  #7 (ethic* OR moral* OR bioethic* OR Jurisprudence OR litigat* OR legal* OR policy OR policies OR law*):ti,ab,kw  #8 MeSH descriptor: [Ethics] explode all trees  #9 MeSH descriptor: [Jurisprudence] explode all trees  #10 MeSH descriptor: [Bioethics] explode all trees  #11 MeSH descriptor: [Morale] explode all trees  #12 MeSH descriptor: [Legislation as Topic] explode all trees  #13 #7 OR #8 OR #9 OR #10 OR #11 OR #12  #14 #3 AND #6 AND #13 | 30 |
| OAIster | kw:("lung cancer" OR "pulmonary cancer" OR "lung neoplasm" OR "pulmonary neoplasm" OR "lung nodule" OR "pulmonary nodule") AND kw:("artificial intelligence" OR "machine learning" OR "deep learning" OR "computer reasoning" OR "machine Intelligence") AND kw:(ethics OR morals OR bioethics OR Jurisprudence OR litigation OR legal OR policy OR law) There is a word number limitation in the search box | 16 |
| CABI | [[All: "lung cancer"] OR [All: "pulmonary cancer"] OR [All: "lung neoplasm"] OR [All: "pulmonary neoplasm"] OR [All: "lung tumour"] OR [All: "lung tumor"] OR [All: "lung nodule"] OR [All: "pulmonary nodule"]] AND [[All: "artificial intelligence"] OR [All: "machine learning"] OR [All: "deep learning"] OR [All: "computer reasoning"] OR [All: "computational intelligence"] OR [All: "machine intelligence"] OR [All: "neural network"] OR [All: algorithm] OR [All: robotics]] AND [[All: ethics] OR [All: moral] OR [All: bioethics] OR [All: jurisprudence] OR [All: litigation] OR [All: legal] OR [All: policy] OR [All: policies] OR [All: law]] | 13 |
